# Enhancing oral squamous cell carcinoma prediction: the prognostic power of the worst pattern of invasion and the limited impact of molecular resection margins

**DOI:** 10.3389/fonc.2023.1287650

**Published:** 2023-12-22

**Authors:** Pavel Hurník, Jana Režnarová, Zuzana Chyra, Oldřich Motyka, Barbora Moldovan Putnová, Zuzana Čermáková, Tomáš Blažek, Martin Fománek, Daria Gaykalova, Marcela Buchtová, Tereza Ševčíková, Jan Štembírek

**Affiliations:** ^1^ Institute of Clinical and Molecular Pathology, University Hospital Ostrava, Ostrava, Czechia; ^2^ Institute of Clinical and Molecular Pathology, Faculty of Medicine, University of Ostrava, Ostrava, Czechia; ^3^ Department of Histology and Embryology, Faculty of Medicine, Masaryk University, Brno, Czechia; ^4^ Department of Oral and Maxillofacial Surgery, University Hospital Ostrava, Ostrava, Czechia; ^5^ Department of Craniofacial Surgery, Faculty of Medicine, Ostrava University, Ostrava, Ostrava, Czechia; ^6^ Department of Hematooncology, University Hospital Ostrava, Ostrava, Czechia; ^7^ Department of Environmental Engineering, VSB-Technical University of Ostrava, Ostrava, Czechia; ^8^ Institute of Animal Physiology and Genetics, Czech Academy of Sciences, Brno, Czechia; ^9^ Department of Pathological Morphology and Parasitology, University of Veterinary Sciences Brno, Brno, Czechia; ^10^ Department of Oncology, University Hospital Ostrava, Ostrava, Czechia; ^11^ Department of Otorhinolaryngology, University Hospital Ostrava, Ostrava, Czechia; ^12^ Institute for Genome Sciences, University of Maryland School of Medicine, Baltimore, MD, United States; ^13^ Department of Otorhinolaryngology-Head and Neck Surgery, Marlene and Stewart Greenebaum Comprehensive Cancer Center, University of Maryland Medical Center, Baltimore, MD, United States; ^14^ Department of Oncology, Sidney Kimmel Comprehensive Cancer Center, Johns Hopkins University, Baltimore, MD, United States; ^15^ Department of Experimental Biology, Faculty of Science, Masaryk University, Brno, Czechia

**Keywords:** orofacial oncology, squamous cell carcinoma, mutation, surgical margins, biomarkers

## Abstract

**Objective:**

Oral squamous cell carcinoma (OSCC) originates from the mucosal lining of the oral cavity. Almost half of newly diagnosed cases are classified as advanced stage IV disease, which makes resection difficult. In this study, we investigated the pathological features and mutation profiles of tumor margins in OSCC.

**Methods:**

We performed hierarchical clustering of principal components to identify distinct patterns of tumor growth and their association with patient prognosis. We also used next-generation sequencing to analyze somatic mutations in tumor and marginal tissue samples.

**Results:**

Our analyses uncovered that the grade of worst pattern of invasion (WPOI) is strongly associated with depth of invasion and patient survival in multivariable analysis. Mutations were primarily detected in the DNA isolated from tumors, but several mutations were also identified in marginal tissue. In total, we uncovered 29 mutated genes, mainly tumor suppressor genes involved in DNA repair including BRCA genes; however none of these mutations significantly correlated with a higher chance of relapse in our medium-size cohort. Some resection margins that appeared histologically normal harbored tumorigenic mutations in TP53 and CDKN2A genes.

**Conclusion:**

Even histologically normal margins may contain molecular alterations that are not detectable by conventional histopathological methods, but NCCN classification system still outperforms other methods in the prediction of the probability of disease relapse.

## 1 Introduction

Malignant head and neck tumors represent approximately 5% of the total number of diagnosed malignant tumors in Europe (1, 2). Squamous cell carcinoma (SCC) is the most common of these tumors, representing more than 90% of all malignant tumors of the oral cavity (MTOC). The etiology of the MTOC is still incompletely understood. The fundamentals include a change of genetic information and cell regulation mechanisms on both the molecular and submolecular levels, leading to uncontrolled proliferation of the affected cell and growth of the tumor mass.

The recurrence of oral squamous cell carcinoma (OSCC) after surgical resection is, unfortunately, common, even in the absence of evident clinical signs of a residual neoplastic tissue. This may be caused by pro-oncogenic events that may have already occurred in the seemingly healthy tissue on the molecular level, which cannot be detected by standard imaging methods (3). Pathological T-status was found to be a good predictive marker for intraoperative additional resection (4); however, finding sensitive biomarkers predicting the recurrence of OSCC or detecting it early could be instrumental in improving the patient prognosis (5) as well as in the personalization of patient management. Several studies have reported that OSCC patients with negative imaging results for metastases may still have micrometastases in the lymph nodes, ranging from 23% to 32%, which can significantly worsen the survival prognosis (6–8). However, the depth of tumor invasion in the biopsy, which is often used as a predictor of metastasis, was not confirmed as a reliable indicator previously (9).

Many factors can contribute to the development of OSCC, including a patient’s lifestyle, oncogenic viruses, or genetic mutations. However, tumors with different etiologies (for example, tumors with different types of genetic mutations) may not display any clinical distinction. This heterogeneity is currently not fully studied, although it may affect the biological behavior of the tumor (10). From this perspective, it could be valuable to have a biomarker indicating the tumor subtype and etiology, which would help individualize the onco-surgical therapy (11). The specificity and sensitivity of such biomarkers are critical in clinical practice. Simplicity and low cost are additional parameters crucial for a biomarker to enter everyday clinical practice (12). With the development of next-generation sequencing (NGS), multi-oncogene panel tests could provide valuable insights for the prognostic and targeted therapeutic approach for OSCC patients; however, it is not routinely employed in clinical practice.

The evaluation of the positivity or negativity of the resection margins with possible distance measurement is a current standard for routine examination of tumor resection. The depth of invasion or ingrowth into the adjacent bone and nodal status constitute the basis for staging and the depth of invasion ratio (MDR) was recently found to be the best predictor of OSCC recurrence (13). Perineural invasion and angioinvasion are additional prognostic factors, while the presence of dysplasia at resection margins was not confirmed to be a risk factor for tumor recurrence or patient survival (14). There are, however, other parameters that are not routinely evaluated, such as the assessment of budding and the worst pattern of invasion (WPOI), which, especially in the early stages of the disease, predict the aggressive behavior of the tumor (15) and may contribute to the assumption of metastasis (16). For these reasons, we focus on these factors in our study.

Biomarkers could also be very useful in evaluating resection margins in surgical therapy. In clinical practice, the resection margins may be histologically “clean” (R0), but the adjacent epithelium in the oral cavity may already contain pro-oncogenic mutations that are not yet clinically evident (12). In such cases, biomarkers could help to determine the frequency of follow-ups or the need for further increase of resection margins (17). Recently, large efforts have been made to evaluate resection margins to assess their surgical safety (for review, see 18). Metabolic perturbations were found in dysplastic margins and several amino acids and lipid ions were identified as markers for the determination of safe surgical margins (19, 20).

To address these challenges, we conducted a comprehensive analysis of multiple histological and molecular features in OSCC patients. First, we performed a retrospective study in OSCC patients aiming to uncover the influence of growth patterns (WPOI, tumor budding) on local cancer recurrence. Using these results, a prospective study was performed to analyze somatic mutations in the tumorous tissues and resection margins as information from European populations is still limited.

## 2 Materials and methods

### 2.1 Patients’ samples

Our study is divided into two parts: retrospective (n=232 OSCC patients) and prospective (n=38 OSCC patients). The inclusion criterion for the study was the diagnosis of SCC in the oral cavity confirmed by biopsy and the indication for tumor resection and block neck dissection. The exclusion criterion was inoperability. All patients included in this study signed an informed consent. The study was conducted in accordance with the current version of the Helsinki Declaration and was approved by the Ethics Committee of the University Hospital Ostrava (No. 514/2018). The retrospective study included patients who underwent radical resection of a biopsy-verified OSCC tumor with synchronous bilateral block dissection of cervical lymph nodes at the Department of Oral and Maxillofacial Surgery at the University Hospital Ostrava, Czech Republic, between 2006 and 2016 (**Table S1**). All cases were retrospectively reviewed, and histopathological variables, such as perineural invasion (PNI) and lymphovascular invasion (LVI), mode of invasion (MOI), the worst pattern of invasion (WPOI), an immune response, tumor budding, and metastasis to ipsilateral or contralateral lymph nodes, were evaluated. Furthermore, TNM reclassification according to AJCC 8th edition was performed, indicating the depth of invasion (DOI). After the procedure, each patient underwent a multidisciplinary team (surgeon, oncologist, pathologist) regarding the following therapy (second look resection, radiotherapy etc.).

**Figure 1:**
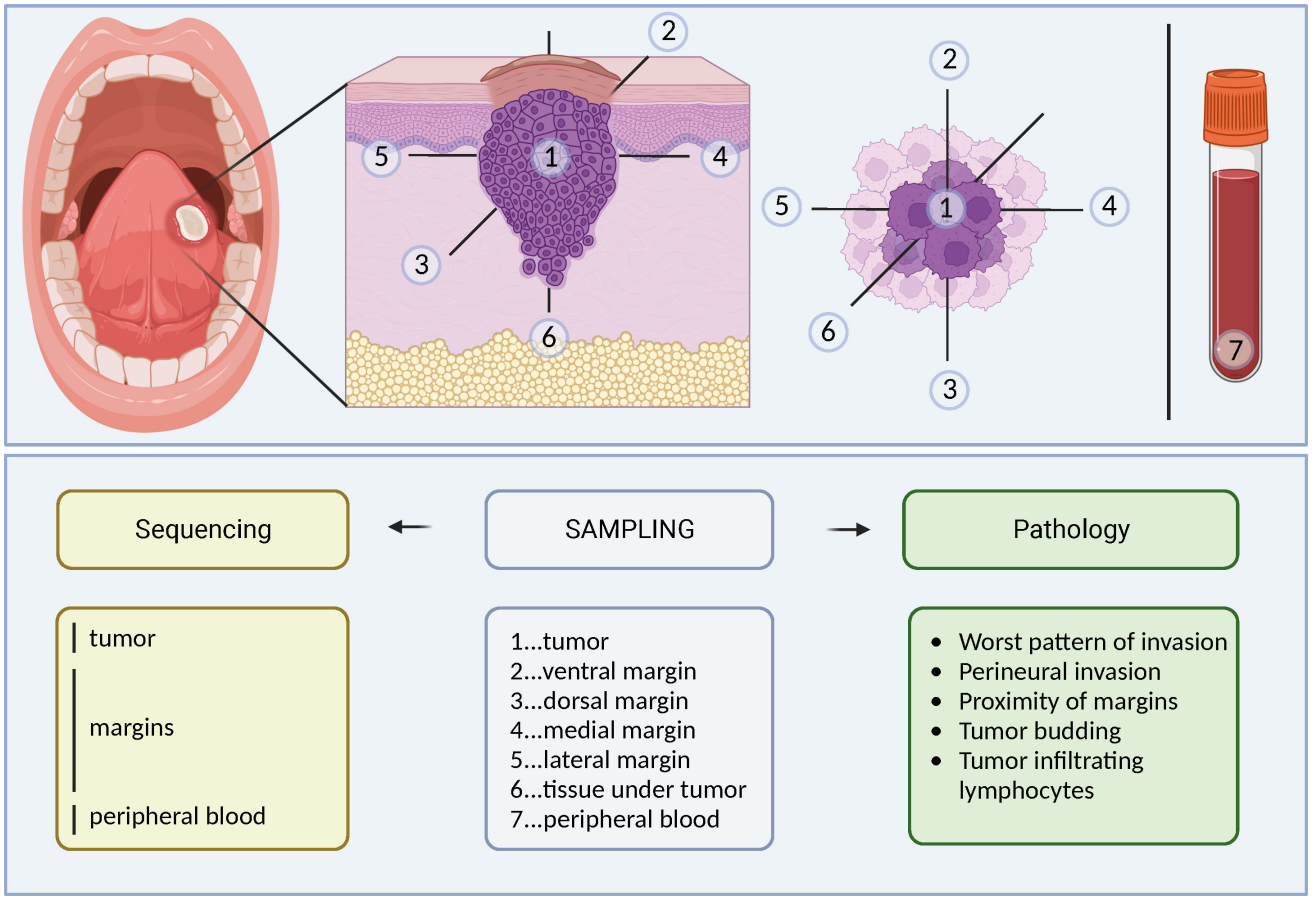
Schema of tissue collection for prospective analysis. Seven tissue samples (1x blood, 1x tumor, 5x resection margins) were collected for histopathological and genetic analysis during clinically indicated radical resection of the tumor with neck block dissection.

The prospective part of the study included patients treated for OSCC at the same department between 2018 and 2020 (**Table S2**). We collected seven samples from each patient (**Figure 1**): one from the tumorous tissue, five from different positions of the seemingly healthy tissue outside the resection margins (i.e., on the outer borders of the wound), and one from peripheral blood (which served as a control for germline mutations). Each tissue sample was snap-frozen immediately in RNAlater (Thermo Scientific, Waltham, MA, USA) after resection. Peripheral blood samples (to exclude congenital polymorphism) were collected in EDTA tubes to prevent coagulation, frozen at -20°C, and transported to the Biobank of the University Hospital Ostrava. Individual samples were registered under a research ID number to guarantee patient anonymity. All samples were used for isolation of DNA/RNA (see below).

### 2.2 Histological processing of tissues and immunohistochemical analysis

**Figure 2:**
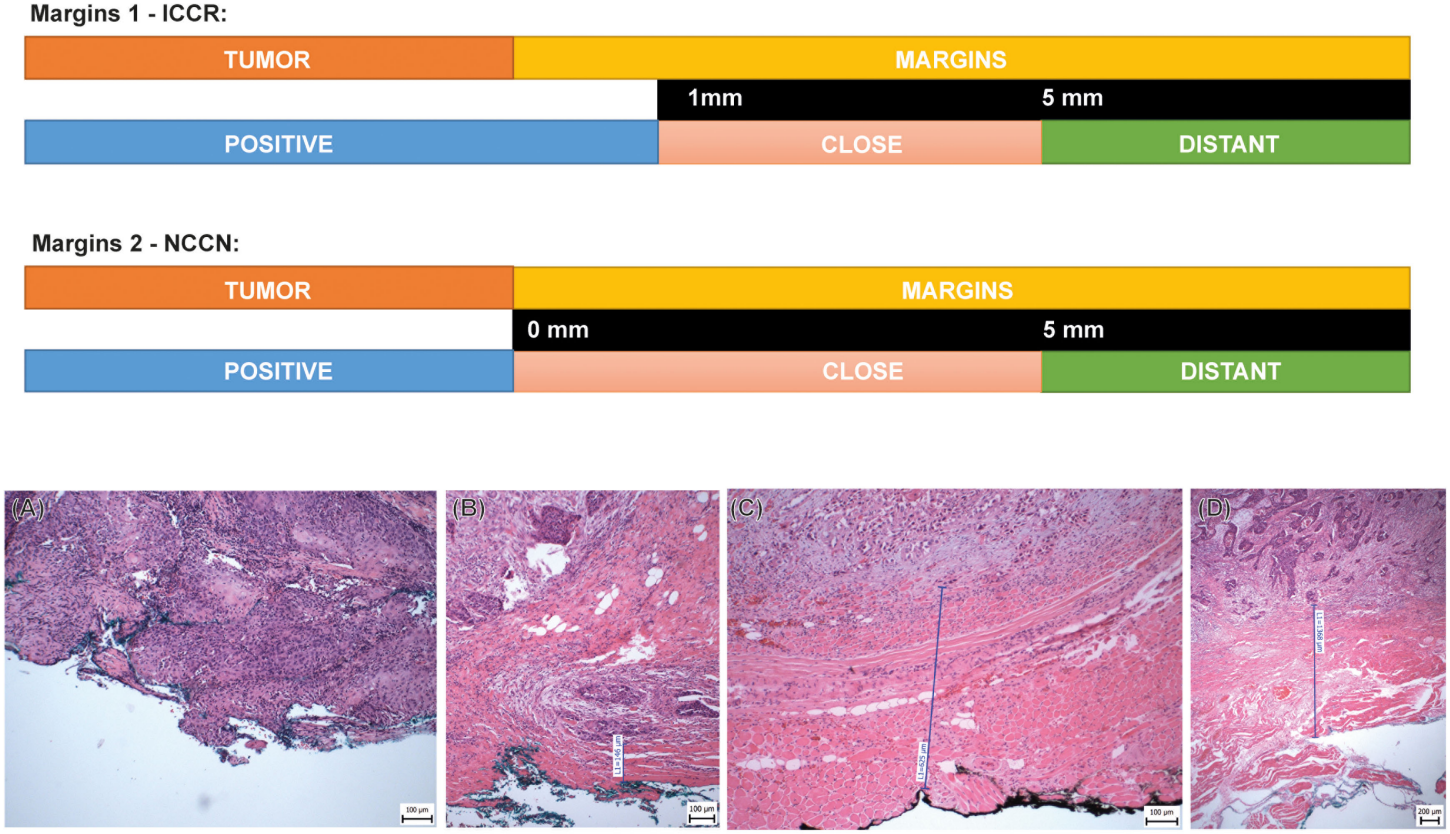
Schema of two various types of histological evaluations of the resection margins. Margins 1 - ICCR: The distance of the tumor ≥ 5 mm is evaluated as a negative/distant margin; close margin is defined as distance from 5 mm to ≥1 mm; positive margin is defined as distance < 1 mm from the resection margin. Margins 2 - NCCN: The distance of the tumor ≥ 5 mm is evaluated as a negative/distant margin, the close margin is defined as the distance from 5 mm to 0 mm, and the positive margin is defined as the actual reaching of the margin. **(A)** Tumor reaching the resection margin, **(B)** Tumor distant 0.15 mm from the resection margin (close/positive margin), **(C)** Tumor distant 0.63 mm from the resection margin (close/positive margin), **(D)** Tumor distant 1.35 mm from the resection margin (close margin), HE.

For histological evaluation, all tissue samples from complete tumor resections were fixed in 4% neutral-buffered formalin. After fixation, the samples were processed by a histopathologist, and representative samples were embedded in paraffin. We cut 4 µm sections from the paraffin blocks and stained them with hematoxylin & eosin (H&E). Reviewing H&E slides, we measured all five margins (four surface margins and one basal/bottom) of each biopsy sample (**Figure 2**). Two scales of evaluation were used: margin evaluation 1 according to the International Collaboration on Cancer Reporting (ICCR) (distant margin ≥ 5 mm, close margin < 5 mm and ≥ 1 mm, positive margin < 1 mm) (21) and margin evaluation 2 according to the NCCN (distant margin ≥ 5 mm, close margin < 5 mm and > 0 mm, positive margin 0 mm) (Head and neck cancers: National Comprehensive Cancer Network (NCCN) guidelines version 3.2021. [May;2021]; https://www.nccn.org/guidelines/guidelines-detail?category=1&id=1437 2021). In all samples, additional morphological parameters, such as the presence of PNI, WPOI (22), tumor budding, and immune response, were also assessed.

Immunohistochemical (IHC) staining was performed according to standard protocols on an automated immunostainer (Ventana BenchMark Ultra, Ventana Medical Systems, Tucson, USA). Sections were cut, deparaffinized, rehydrated, and pre-treated with an antigen retrieval buffer (Tris/Borat/EDTA, pH 8.4). After blocking of endogenous peroxidase, the slides were incubated with monoclonal antibodies directed against p16 (clone R15-A, DB BIOTECH, dilution 1: 200), p53 (clone NCL-L-p53-DO7, Leica Novocastra, dilution 1: 200), and cytokeratin (clone AE1/AE3, Zytomed Systems, dilution 1 : 200). All tissues collected for prospective analyses and mutation screen (n = 38) underwent IHC analysis for p16 detection with a negative result.

### 2.3 Isolation of DNA for sequence analysis

RNA and DNA from snap-frozen tumor tissues that were stored in RNAlater were isolated using an Allprep DNA/RNA kit (Qiagen, Hilden, Germany). DNA from resection margins (samples numbered 2–6, **Figure 1**) were pooled equimolarly. We extracted DNA also from the peripheral blood using a MagCore automated extractor (Anatolia Geneworks, Istanbul, Turkey). The quality and quantity of DNA were measured using a Nanodrop 2000 spectrophotometer (Thermo Scientific) and a Qubit 2.0 fluorometer (Invitrogen, Carlsbad, CA, USA). The quality control (QC) checks of DNA for library preparation were performed using electrophoresis on 1% agarose gels and a Qubit dsDNA HS Assay Kit with a Qubit 2.0 fluorometer (Life Technologies, Carlsbad, CA, USA) according to the manufacturer’s instructions.

### 2.4 Generation of libraries for NGS

Libraries were prepared with the SureSelect XT protocol (Agilent Technologies, Santa Clara, CA, USA) with Axen™ Cancer Panel 1 (88 genes; **Table S3**) developed by Macrogen (Seoul, Korea). The quality of the libraries was checked using a 2100 Bioanalyzer (Agilent Technologies) to verify the product ranged from 200 to 400 bp. Then, the libraries were quantified using a Qubit dsDNA HS Assay Kit and Qubit 2.0 fluorometer (Life Technologies, Waltham, MA, USA). The libraries were subjected to paired-end sequencing (2 × 150 bp) on a NextSeq500 instrument (Illumina, San Diego, CA, USA) using high output mode and sequencing by synthesis chemistry.

### 2.5 Variant calling in NGS data

The adapter sequences were removed by fastp (23). Trimmed reads were aligned to the reference genome (GRCh37/hg19) using BWA-MEM (24). Poorly mapped reads with a mapping quality (MAPQ) below 20 were removed using Samtools version 1.3.1 (25). Duplicated reads were discarded using Sambamba markdup (version 0.6.7) (26). The base quality of deduplicated reads was recalibrated using GATK BaseRecalibrator. Somatic mutations, including single nucleotide variants (SNVs) and small insertions and deletions (INDELs), were identified using the MuTect2 algorithm (27). We discarded mutations with a variant allele frequency (VAF) or a depth lower than 2% or a total depth lower than 100×. We also excluded variants with a minor allele frequency (MAF) of ≥ 1% in genomA-D and ExAC. All the remaining variants were annotated using SnpEff and SnpSift v4.3i (28).

### 2.6 Filtration of variants for tumor/margin tissue

Illumina sequencing was performed by using a panel of 88 cancer genes (Axen Cancer Panel I, containing tumor suppressors and oncogenes, **Table S3**) and aimed for sequencing coverage of about 2,000× per sample. For further analysis, only non-synonymous variants in tumor/margin that were classified as “pathogenic”, “likely pathogenic”, “uncertain significance”, or “conflicting interpretations of pathogenicity”, in the ClinVar database and at the same time were absent in the peripheral blood (germline mutations) were selected.

### 2.7 Variant validation by Sanger sequencing

As mentioned above, total RNA was isolated from the same sample using an Allprep DNA/RNA kit (Qiagen). The RNA quality and quantity were measured using a Nanodrop 2000 spectrophotometer (Thermo Scientific) and a Qubit 2.0 Fluorometer (Invitrogen). RNA was transcribed to cDNA using a ProtoScript II First Strand cDNA Synthesis Kit (NEB, UK). cDNA was used for amplification to confirm the presence of mutations detected by Axen™ Cancer Panel 1 (Macrogen) in the DNA of patient samples. Gene-specific primers were designed by us using the NCBI primer design tool (https://www.ncbi.nlm.nih.gov/tools/primer-blast/). PCR products were visualized on 1% agarose gels using electrophoresis and purified using a QIAquick PCR Purification kit (Qiagen, Germany). Purified products were sequenced by Sanger sequencing with the amplification primers (Eurofins Genomics, Ebersberg, Germany). Obtained sequences were mapped to the reference sequences from GenBank. To validate the mutations in the margins, we tested RNA from individual margins separately, without pooling, unlike for the gene panel NGS sequencing.

### 2.8 Gene expression analysis by qPCR

We performed gene expression analysis on selected tissue samples with sufficient quality and quantity of mRNA. The total mRNA was transcribed to the cDNA using an Elite Reverse Transcription Kit (Generi Biotech, Hradec Kralove, Czech Republic).

Gene expression analysis of CDKN1A (Hs00355782_m1), CDKN2A (Hs00923894_m1), BAX (Hs00180269_m1), PUMA (Hs00248075_m1), and GAPDH as a housekeeping gene (Hs02758991_g1) was performed using LightCycler 96 (Roche, Mannheim, Germany) during 40 cycles of 95°C/15 s and 60°C/1 min, using TaqMan™ Gene Expression Assays (Thermo Fisher Scientific, USA).

Gene expression levels were calculated using ΔCt analysis with normalization to the level of the housekeeping gene GAPDH. To compare gene expression between sample groups, unpaired and paired two-tailed Student’s t-tests were performed in GraphPad (GraphPad Software Inc., San Diego, CA, USA). The differences in expression were considered significant at p < 0.05.

### 2.9 Statistical assessment

All statistical analyses and visualizations were performed in the R environment (29). Comparisons of two groups’ means were assessed using the Welch two-sample t-test; three and more groups were assessed by one-way ANOVA. Factorial analysis of mixed data (FAMD) and consequent hierarchical clustering on principal components (HCPC) were performed in the R packages FactoMiner (30) and factoextra (31), respectively. Survival analyses were performed using the R package survival (32). Multivariable Cox proportional hazard model was calculated using DATAtab online software (Graz, Austria) from variables that proved statistical significance in univariable analysis in the corresponding dataset (retrospective and prospective) in this paper.

## 3 Results

### 3.1 Retrospective analysis of OSCC patients: dependence of histological and molecular features with survival outcomes

**Figure 3:**
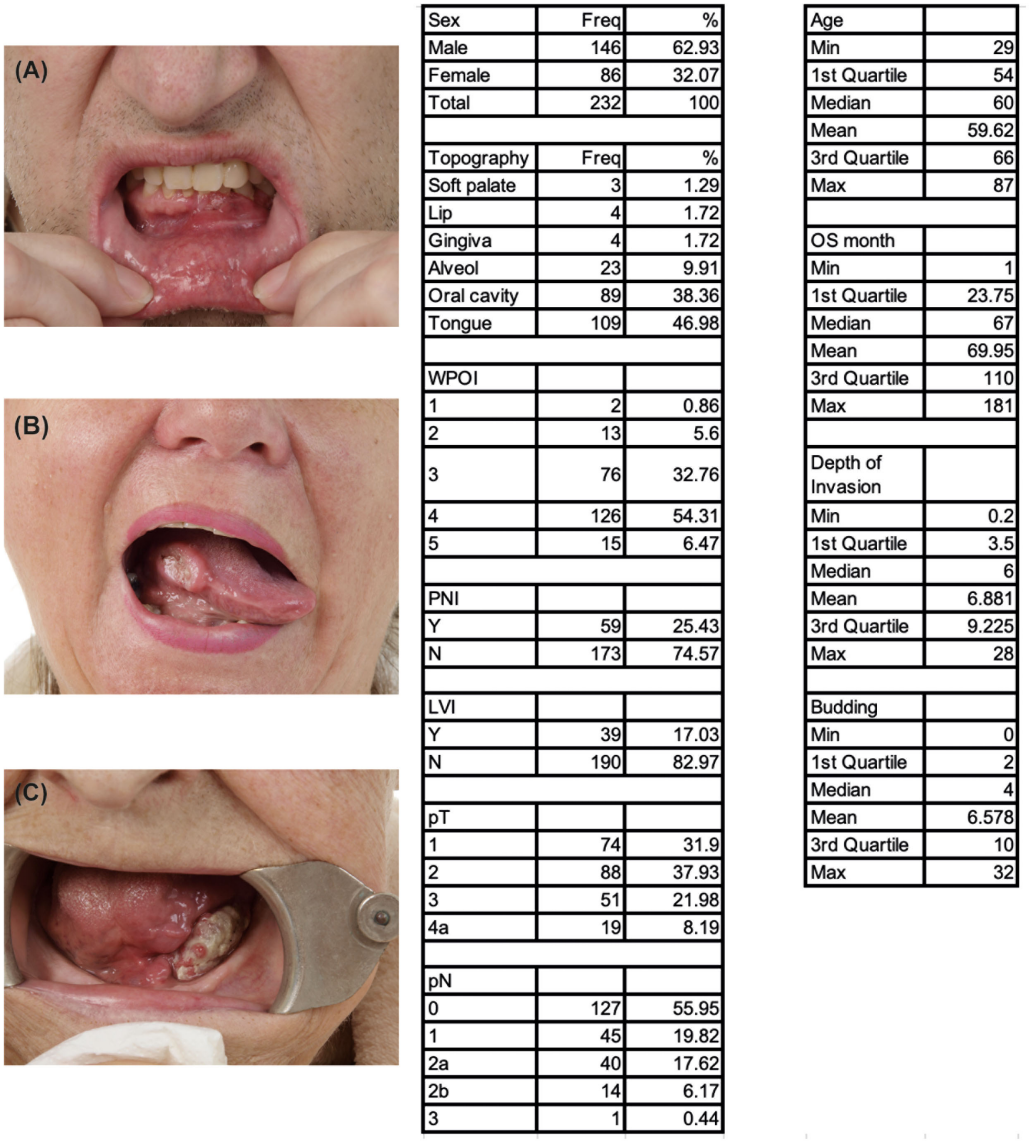
Main morphological features of prospective OSCC patients (n = 232). **(A)** Lower jaw gingival tumor, **(B)** Tumor located on the right edge of the tongue, **(C)** Tumor located on the left side of the oral cavity. Tables display an overview of key morphological features of the prospective cohort (n=232 patients).

First, we conducted a retrospective study of 232 OSCC patients (**Figure 3**) of which 146 (62.93%) were men and 86 (37.07%) women, aged 29–87 years (median 60). The most common OSCC location was the tongue (109 cases; 46.98%), followed by the oral floor (89 cases, 38.36%), alveolus of the jaw (23 cases, 9.91%), mucosal side of the lip and gingiva (4 cases each, 1.72%), and the soft palate (3 cases, 1.29%). PNI was present in 59 patients (25.43%) and absent in 173 patients (74.57%). We measured the DOI of OSCC, evaluated its association with PNI, WPOI, budding, and metastasis, and performed a cluster analysis to identify groups of patients with different characteristics and outcomes.

### 3.2 The depth of invasion is dependent to PNI and the occurrence of metastasis

**Figure 4:**
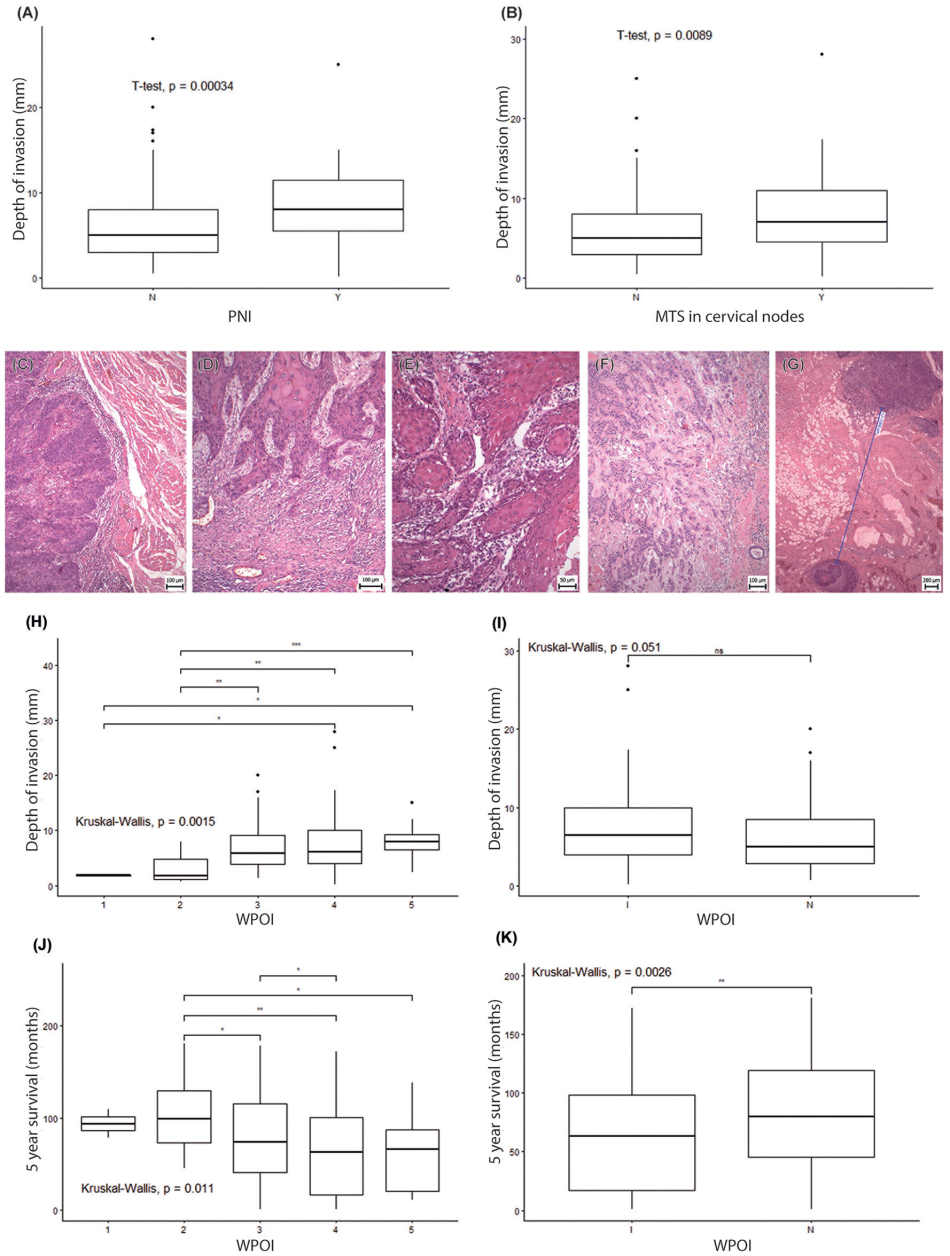
The worst pattern of invasion in OSCC patients. **(A, B)** Boxplots display the association of the depth of invasion on the PNI status **(A)** and metastases occurrence in cervical nodes **(B)**. In both cases, the difference was statistically significant based on the t-test. Representative microphotographs of the worst pattern of invasion (staining HE). **(C)** WPOI1 – Typical pushing border, **(D)** WPOI2 – Finger-like growth pattern, **(E)** WPOI3 – Large separate islands, containing more than 15 cells, **(F)** WPOI4 – Small tumor islands, containing less than 15 cells per island, **(G)** WPOI5 – A tumor with satellites that are ≥ 1 mm from nearest satellite or the tumor itself. **(H)** Association analyses of separate WPOI types to DOI by Kruskal-Wallis test. **(I)** Kruskal-Wallis test associating “infiltrative pattern” of invasion "I" and “non-infiltrative pattern” of invasion "N" to DOI. **(J)** Kruskal-Wallis test associating separate WPOI types with 5 year survival. **(K)** Kruskal-Wallis test associating “infiltrative pattern” of invasion "I" and “non-infiltrative pattern” of invasion "N" to 5 year survival. * - p<0.05, **- p<0.01, ***- p<0.001. ns, not significant.

DOI was measured retrospectively in all cases as the distance from the basement membrane “horizon” of the tumor-adjacent mucosa to the deepest point of tumor invasion. The DOI values ranged from 0.2 to 32 mm, with a median of 4 mm. DOI was found to be dependent on tumor metastasizing, PNI, WPOI and budding. As evidenced by Welch two-sample t-test, (**Figure 4A**, p = 0.00034), the effect of PNI on DOI is prominent. Occurrence of lymph node metastasis was, similarly, found to have an effect on DOI (**Figure 4B**, p = 0.0089). This suggests that DOI is a useful indicator of tumor aggressiveness and metastatic potential.

### 3.3 Association of worst pattern of invasion grade with DOI and patient survival

Where WPOI is concerned (**Figures 4C–G**), 0.86% (2/232) patients displayed WPOI Grade 1; 5.6% (13/232) exhibited WPOI 2; 32.76% (76/232) WPOI 3; 54.31% (126/232) WPOI 4; and 6.47% (15/232) corresponded to WPOI 5 (**Table S1**). We performed a statistical analysis to examine the associations between the DOI and the WPOI grades and found a significant difference (p = 0.0412), with maximum differences between WPOI Grade 4 and Grade 2 and between WPOI Grade 5 and Grade 2 (**Figure 4H**, p = 0.0015). If we merge WPOI 1-3 into the group “non-infiltrative pattern” of invasion and WPOI 4-5 into the group “infiltrative pattern” of invasion, the Kruskal-Wallis test uncovered a very close association with the depth of invasion (**Figure 4I**, p = 0.051). We also detected a significant difference (p = 0.0112) in survival outcomes for different WPOI grades, with the lowest survival rate for WPOI Grade 4 and the highest for WPOI Grade 2 (**Figure 4J**). The “infiltrative pattern” of invasion was also associated with shorter survival than the “non-infiltrative pattern” (**Figure 4K**, p = 0.0026). This data suggests that WPOI is a significant factor influencing both DOI and survival, as higher WPOI grades are associated with higher DOI and worse survival. “Infiltrative pattern” WPOI was also confirmed as an independent predictor in a multivariable analysis, similarly as the presence of cervical metastasis and DOI. Interestingly, among other predictors, WPOI reached the highest hazard ratio (HR=1.64, CI 1.01 - 2.67) in our analysis (**Table S4**).

### 3.4 Tumor budding negatively correlates with patient survival

**Figure 5:**
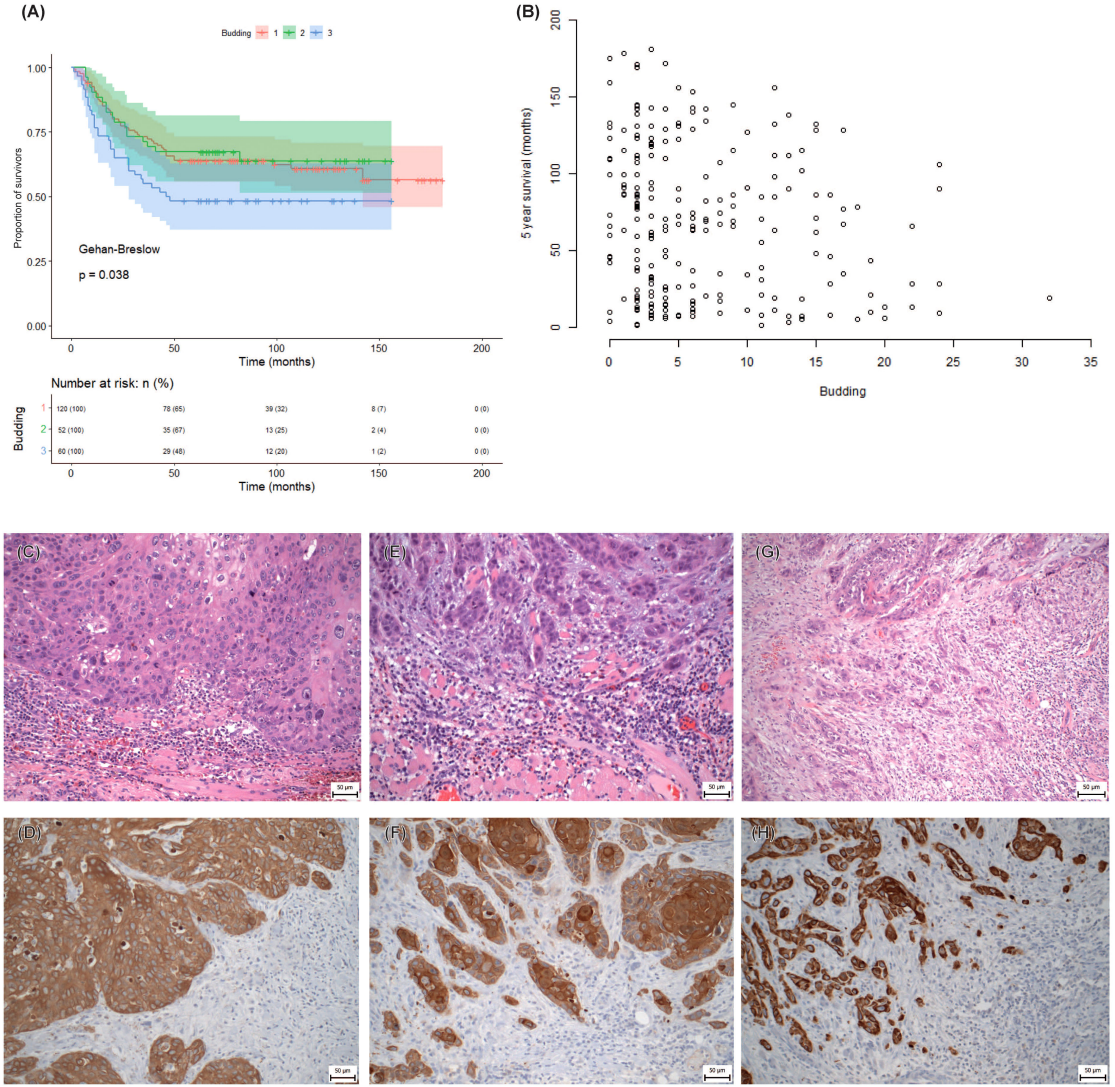
Tumor budding in the retrospective study. **(A)** Kaplan-Meier survival curve of three groups of patients divided according to the tumor budding observed. **(B)** Overall reliance of the patient’s survival on the tumor budding. **(C–H)** Representative microphotographs of tumor bud abundance. **(C, E, G)** Staining by Hematoxylin-Eosin. **(D, F, H)** Immunohistochemical detection of pan-cytokeratin AE1/AE3 to visualize epithelial tumor buds, x200 magnification. Cytokeratin-positive tissue (DAB, brown), cytokeratin-negative tissue (Hematoxylin, blue). **(C, D)** Low intensity of tumor budding (< 5 buds). **(E, F)** Intermediate intensity of tumor budding (>5 buds - < 10 buds). **(G, H)** High intensity of tumor budding (≥ 10 buds).

We also examined the effect of tumor budding on 5-year survival and found a significant negative correlation (R= −0.202, p = 0.002) (**Figure 5**). We classified the tumors into three groups based on the number of buds per high-power field (HPF): group 1 (1–5 buds/HPF), group 2 (6–10 buds/HPF), and group 3 (>10 buds/HPF). We compared the survival rates among these groups and found that group 3 had the lowest survival rate (p = 0.038). This indicates that high-grade budding is a predictor of poor prognosis for OSCC, nevertheless cannot serve as an independent predictor of prognosis as revealed in multivariable analysis in retrospective dataset with respect to DOI, WPOI or metastasis, despite its promising results from a smaller prospective dataset (chapter 3.6; **Table S4**).

### 3.5 Hierarchical clustering of risk groups

**Figure 6:**
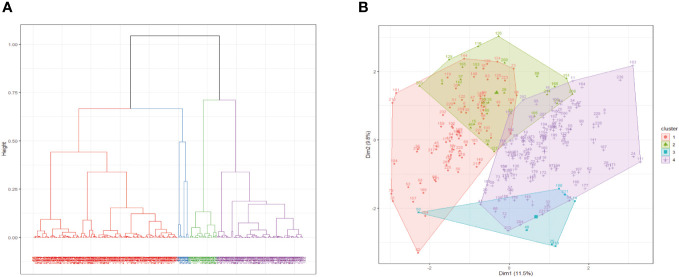
Hierarchical clustering based on principal components. **(A)** Dendrogram of the clusters resulting from the Hierarchical clustering on principal components. **(B)** Resulting clusters superimposed on the FAMD first factor plane.

We applied hierarchical clustering on the principal components obtained from FAMD to group the patients based on their similarities (**Figure 6**). Based on the inertia gain analysis, we determined the optimal number of clusters to be 4.

Topography, WPOI, 5-year survival, sex, budding, age, and DOI were found to be the determining variables (**Figure 6**). Cluster 1 was characterized by less budding, soft palate localization of the tumor, WPOI 2 and 3, survival for more than 5 years, and male sex. Cluster 2 was characterized by higher mean age and lower DOI as well as by tumor localization predominantly in the dental alveolus and lip, with rare localizations on the oral floor and tongue. Cluster 3 was mainly defined by gingival tumors. Cluster 4 exhibited high budding, high DOI, lower mean age, oral cavity tumor, less than 5-year survival, and WPOI 4 and 5, while alveolar tumors and WPOI 2 and 3 were uncommon.

We also aimed to investigate morphological factors that are not routinely assessed by histopathological evaluation and to combine them with established factors to identify a group of high-risk patients or a factor that can indicate the aggressiveness of the disease. We found that DOI and WPOI (especially its correlation between low- and high-grade morphological types) were statistically significantly associated with the outcomes. In the same way, tumor budding was a negative morphological prognostic factor, as high-grade budding morphology was associated with a high-risk patient group, which was confirmed by our statistical analysis.

In summary, in the retrospective part of the study, we identified four groups of patients, where the group of high-risk patients (Cluster 4) was characterized by WPOI 4 and 5, tumors located in the floor of the oral cavity, increased budding, higher DOI, and younger age.

### 3.6 Budding of OSCC and margins as markers of future tumor behavior

Next, we aimed to examine the significant variables of the retrospective dataset in relation to the margin status. However, the margins in the retrospective dataset were classified only as positive or negative. Therefore, we wanted to identify the margin features that could predict tumor recurrence or early death. We selected several patients for further prospective analysis, in which the resection margins were measured in detail. We applied two models of margin distance assessment (described in Material and Methods). We evaluated the above-mentioned variables and their correlations with survival, recurrence, and margin status in these patients.

The tumor outgrowth is characterized by a tree-like appearance with numerous branching and lateral projections, with tumor cells connected at the base and a very complex 3D structure, similar as published for other carcinomas (33). However, the tumor is composed only of sheets of neoplastic cells; nevertheless, the tissue becomes discohesive in the periphery, and detached clusters of neoplastic cells form small tumor buds. Tumor budding was the most significant risk factor in our retrospective analysis. Since it occurs at the tumor periphery, we questioned whether the evaluation of the margins as positive/close/distant was adequate. Tumors with high-grade budding displayed more frequent recurrence. The tumor’s ability to form small epithelial islands or dissociated isolated tumor cells opens up the possibility that genetic alterations are already present in the microscopically normal tissue and contribute to the tumor’s aggressiveness or recurrence.

**Figure 7:**
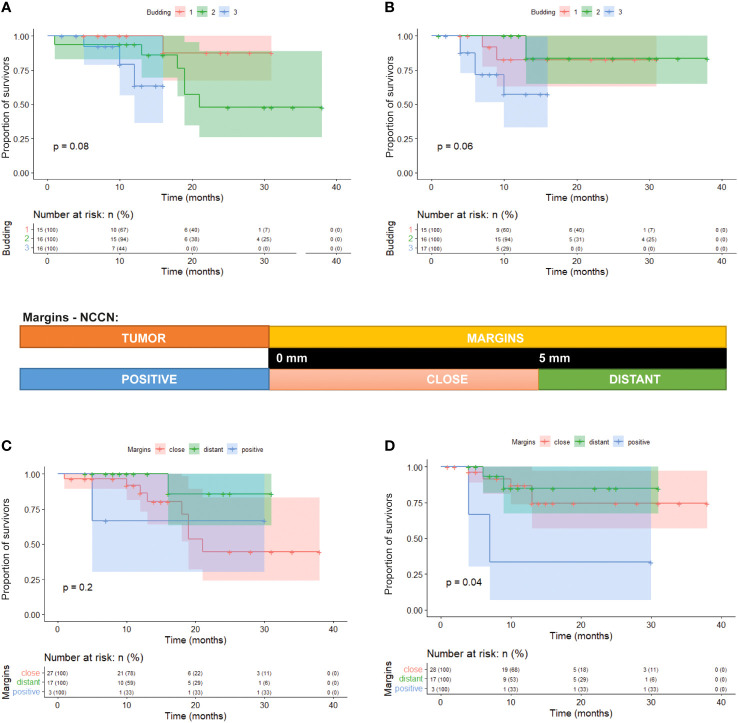
Kaplan-Meier survival curves of three groups of patients divided according to the tumor budding. **(A)** Tumor budding vs overall survival (p=0.08). **(B)** tumor budding vs recurrence of the tumor (p=0.06). **(C)** Correlation of NCCN margin evaluation vs overall survival (p=0.2). **(D)** Correlation of NCCN margin evaluation vs recurrence of the tumor (p=0.04).

From the morphological point of view, we found a negative correlation between tumor budding and disease recurrence in the prospective study. Margin positivity according to the ICCR criteria was not associated with survival or disease recurrence (TTP), while margin positivity according to the NCCN criteria was significantly associated with recurrence but not with overall survival (**Figure 7**, **Figure S1**). Analysis of the impact of mutation in the margin yielded a slightly higher occurrence of relapse in patients with mutated margins (p = 0.102), though the overall survival was not affected at all (p = 0.66). Multivariable analysis of parameters describing tumor margins suggested both the budding and NCCN could be independent variables, however some other parameters were not tested or insignificant in univariate analysis in this small dataset (DOI, WPOI, PNI, metastases) (**Table S4**).

### 3.7 Molecular profiling of tumor and margin samples using a cancer gene panel

Furthermore, we explored the pathological features of tumor margins that might not be detected by histopathology and to identify potential prognostic markers. We also wanted to investigate the molecular mechanisms involved in tumor budding or possible alterations in molecular signaling in the adjacent tissue, as well as to find markers of shorter TTP (time to progression).

**Figure 8:**
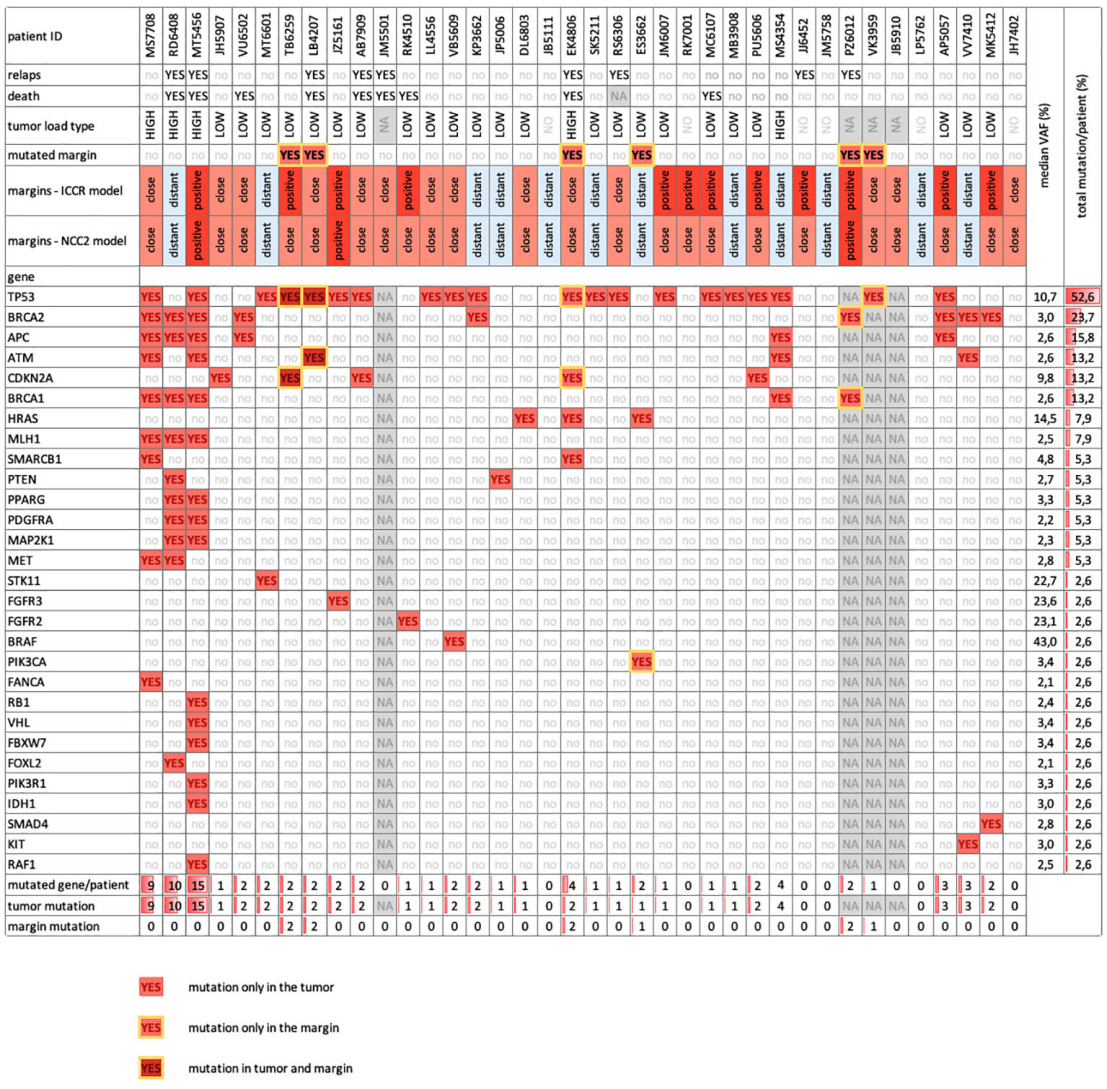
Overview table depicts basic clinical parameters and mutated genes captured within the prospective patient cohort by NGS (n = 38). We detected the mutations in 30 different genes (rows) in n=38 prospective patients with OSCC (columns) and their resection margins. We recorded the clinical parameters of the patients and classified them according to their mutation load. The mutation load was defined as the number of mutated genes per patient, ranging from 0 to 15 per patient. We considered a mutation load of 1-3 as low and a mutation load of 4-15 as high. The most frequently mutated genes were *TP53* (52.6%), *BRCA2* (23.7%), and *APC* (15.8%). We observed three patterns of mutation distribution: genes that were mutated only in tumor tissue (light red), genes that were mutated in both tumor and margin tissue (dark red and yellow border) and genes that were mutated only in margin tissues in a respective patient (light red with yellow border). There were five genes (*TP53, CDKN2A, BRCA2, BRCA1, PIK3CA*) that belonged to the last category. Six patients had mutations in their resection margins.

**Figure 9:**
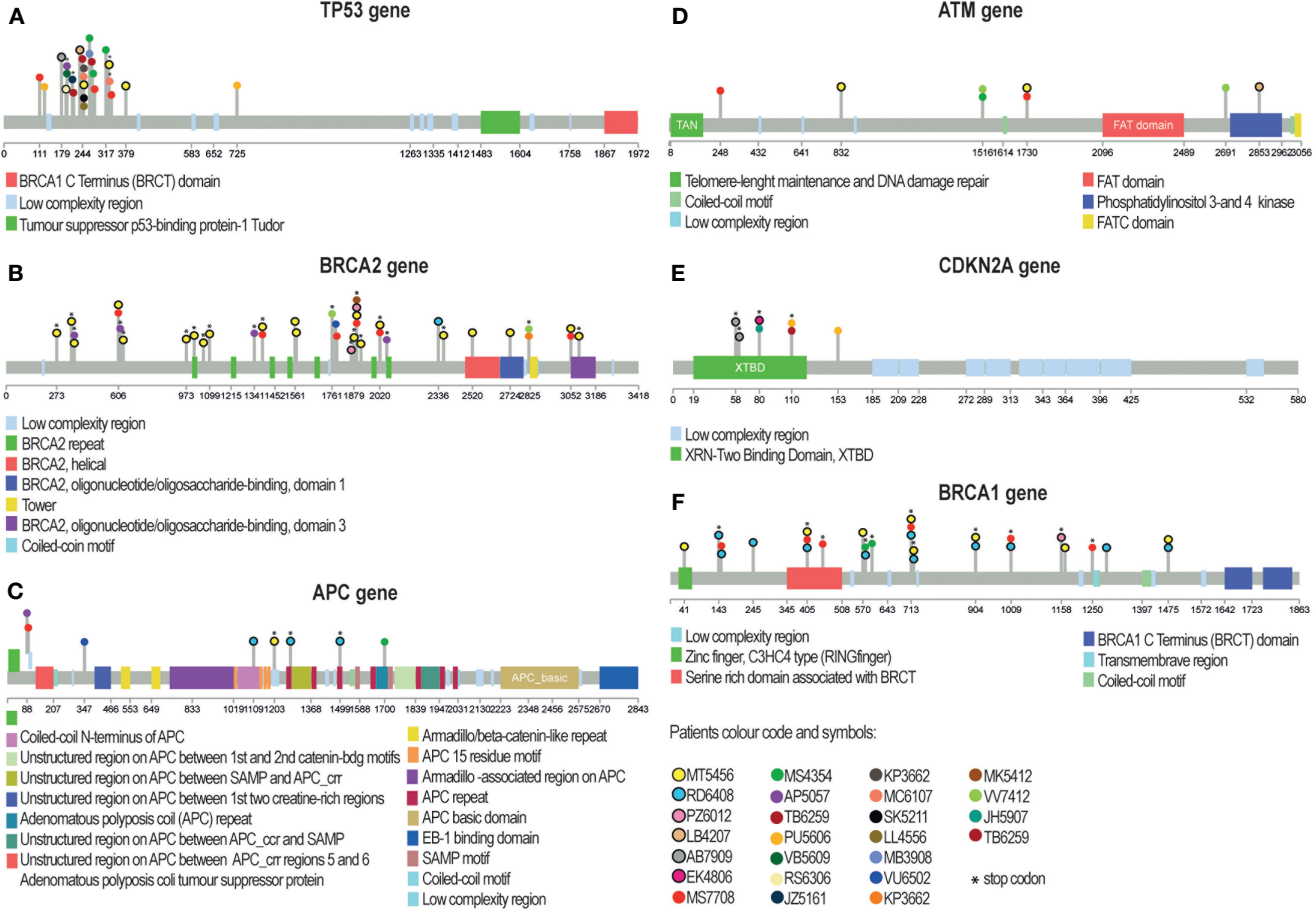
Lollipop plots showing somatic mutations in the six most frequently mutated genes in our patient cohort. Mutations are shown for TP53 **(A)**, BRCA2 **(B)**, APC **(C)**, ATM **(D)**, CDKN2A **(E)**, and BRCA1 **(F)** genes. The colored boxes indicate specific functional domains of the proteins. Each circle represents a mutation in a patient and has a color corresponding to the patient ID. Circles with a black border indicate mutations in margin tissue. Asterisks mark mutations that cause premature stop codons, which were common in genes BRCA2 **(B)**, CDKN2A **(E)**, and BRCA1 **(F)**. A) TP53 mutations occurred in 20 patients and were primarily located in the trans-activation and proline-rich domains. E) CDKN2A had almost all mutations clustered in the binding domain. **(B)** BRCA2 and BRCA1 **(F)** had several mutation hotspots in our cohort. BRCA2 **(B)**, APC **(C)**, ATM **(D)**, and BRCA1 **(F)** had mutations distributed along the entire coding region.

DNA was isolated from 38 patients (however, four patients had incomplete sample sets, as indicated in **Table S2**). We utilized peripheral blood as a control for germline mutations. In the tumor tissue, we detected a total of 108 variants in 29 genes in 28 out of 34 sequenced tumor samples (**Figure 8**; **Table S5**). Conversely, we were not able to identify any mutation in six tumor samples. We identified a median of 1.5 mutations per tumor (range 0–15). The median Variant Allele Frequency (VAF) was 3% (range 2.1–43%). *TP53* (52.6%), *BRCA2* (23.7%), and *APC* (15.8%) were the three most commonly mutated genes. The exact positions of mutations in these mutated genes are provided in **Figure 9**. Fourteen genes were mutated in more than two tumors.

Eleven variants in six genes were shared between tumors and margins (**Figure 9**; **Table S6**) in six patients. The most commonly found mutations in margins were in genes *TP53* and *CDKN2A*. Variant allele frequency was in the range of 3–11%. Thus, some morphologically clean resection margins revealed the presence of tumorigenic mutations.

**Figure 10:**
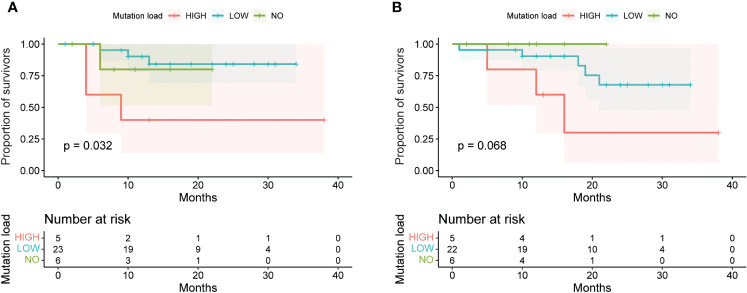
Survival analysis of OSCC patients based on mutation status in tumors and margins. The survival curves were generated by the Kaplan-Meier method and compared by the Log-rank test. **(A)** Patients with high mutation load (more than 4 mutations detected in tumors or margins) had a significantly lower time to progression (TTP) than patients with low mutation load (less than or equal to 4 mutations) or no mutations (p = 0.032). **(B)** However, there was no significant difference in overall survival (OS) among these three groups of patients (p = 0.068).

We correlated the presence of gene mutations in tumor samples with TTP and overall survival and found significant correlation of high tumor load for TTP and not for OS (**Figures 10A, B**). The presence of the mutation in none of the five most frequently mutated genes correlated with TTP, nor with OS. Notably, the results could have been affected by a short follow-up in some patients (less than 6 months in 8 cases).

### 3.8 Confirmation of NGS data by Sanger sequencing

To validate some of the variants detected by NGS, we performed Sanger sequencing. We chose the gene TP53, which had the highest number of mutations, and used RNA as an independent source of mutations. In all tested cases, the *TP53* gene was expressed in tumorous tissue. Sanger results confirmed the presence of mutation in case VAF was higher than 10% by NGS. The presence of mutation was confirmed in four tumor mutations and five margin mutations (**Figure S2**). When the VAF was lower, we could not detect the mutation by Sanger sequencing. Thus, *TP53* mutations can be detected in RNA as well as DNA. Sanger sequencing can confirm the presence of high-VAF mutations detected by NGS but may miss low-VAF mutations.

We also observed that most of the margin mutations were detected in the lateral margin (margin position #5, **Figure 1**) in the three patients. Mutations in ventral and dorsal margins were observed only once. One case had mutations in both lateral and dorsal margins, while the rest of the margin mutations were detected in different patients. This suggests that margin mutations are more common in the lateral margin than in the ventral or dorsal margins and are mostly patient-specific.

### 3.9 Protein expression categories of p53 do not correlate with the occurrence of mutations in the *TP53* gene

Knowing that many missense mutations in *TP53* lead to the gain of function of its protein, we aimed to determine if the mutated *TP53* captured by sequencing is in concordance with aberrant expression of TP53 by IHC (**Table S7**). By IHC, the expression of the “wild-type” TP53 is characterized by nuclear positivity of variable intensity in usually less than 50% of cancer cells. Aberrant expression, pointing to mutations in the *TP53* gene, is typically characterized by intense nuclear positivity in more than 75% of cancer cells or by full negativity of cancer cells (especially in the case of nonsense or frameshift mutations). In the case of full negativity, it is important to find cells with sporadic positivity in non-cancerous tissue, which serves as an internal control.

We evaluated the concordance between the *TP53* status by IHC and by sequencing. The correlation was very low, only 0.052. Given the fact that the *TP53* expression detected by IHC or by sequencing method did not correlate with survival (**Table S8**), we could not determine which approach to the *TP53* mutant status detection was better (**Table S7**). The observed differences could be due to the variable impact of mutations. Instances where mutations were classified as “conflicting interpretations of pathogenicity” or “uncertain significance” were denoted as wild-type by IHC, suggesting their limited influence on protein function. Overall, we found a very low concordance between the two methods and no correlation between *TP53* and survival.

### 3.10 Analysis of *TP53* downstream target genes in relation to *TP53* mutation status and tumor mutation load

**Figure 11:**
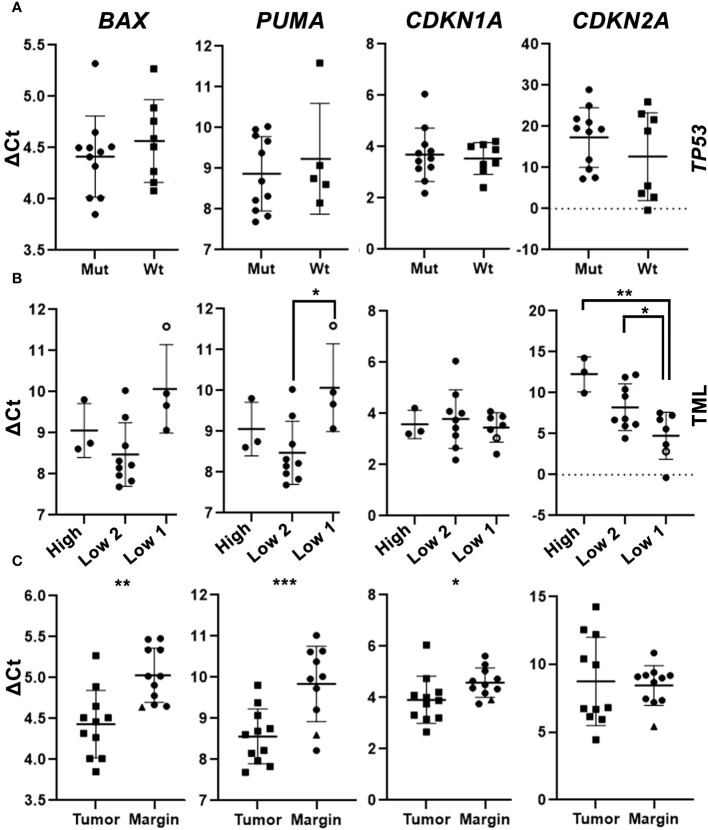
mRNA expression levels of TP53 downstream target genes in the neoplastic and marginal tissue of OSCC. **(A)** mRNA expression of four candidate genes in tumor samples with a detected mutation in TP53 (Mut) and wildtype TP53 (Wt). **(B)** mRNA expression of selected genes in the neoplastic tumor tissue categorized based on the Tumor mutation load (TML): tumors with >2 mutations (High), tumors with 2 mutations (Low 2), and tumors with ≤ 1 mutation (Low 1), the one sample without a detected mutation is depicted as “0”. **(A–C)** Gene expression levels were calculated using ΔCt analysis with normalization to the level of housekeeping gene GAPDH. **(C)** mRNA expression of four genes in tumor samples and margin samples. The only marginal sample with a detected mutation in TP53 and CDKN2A is shown as “▲”. Unpaired t-tests **(A, C)** and paired t-tests **(B)** were used for statistical analysis. * (p <0.05); ** (p <0.01); ***- p<0.001.

Since *TP53* is the most frequently mutated gene, we wanted to analyze in detail the alterations in the expression of downstream targets of this gene (34) in the portion of samples from the prospective part of this study. We used qRT-PCR methodology to detect the expression of four *TP53* target genes (*PUMA, BAX, CDKN1A, CDKN2A*) and their expression was evaluated on paired tumor and margin samples from 21 OSCC patients. Interestingly, we did not find a statistically significant difference in the mRNA expression of *TP53* downstream target genes between tumors with a mutation in *TP53* and tumors with wild-type (Wt) *TP53*. It is important to note that tumors without *TP53* mutation had mutations in (many) other genes (**Figure 11A**).

We found a correlation between tumor mutation load and mRNA expression of *TP53* downstream target genes. For example, the tumors with a low mutation load had a lower expression of *PUMA*, a pro-apoptotic gene. The only tumor sample (*JB5111*) with no detected somatic mutations exhibited the lowest expression of *PUMA* relative to other analyzed samples. Another sample (*JH5907*) with no detected somatic mutation in *TP53* displayed the lowest expression of *BAX* and *PUMA* relative to other analyzed samples, while the expression levels of *CDKN1A* and *CDKN2A* genes in this sample were relatively high (**Figure 11B**). We have also noted that the higher was the tumor mutation load, the lower was the expression of *CDKN2A*. We found that tumors with *TP53* mutations or high mutation load had lower expression of pro-apoptotic and senescence genes than tumors with wild-type *TP53* or low mutation load.

We compared the expression of the *BAX, PUMA, CDKN1A*, and *CDKN2A* genes in neoplastic and non-neoplastic marginal tissues. We found that the pro-apoptotic genes *BAX* and *PUMA*, as well as the senescence gene, *CDKN1A*, were more expressed in the neoplastic than in the non-neoplastic marginal tissues. One of the margin samples (*TB6259*) containing *TP53* and *CDKN2A* mutations displayed high expression of all examined genes. Expression of *CDKN2A* did not differ significantly between neoplastic and non-neoplastic tissues (**Figure 11C**). We also found that neoplastic tissues had higher expression of pro-apoptotic and senescence genes than non-neoplastic marginal tissues, except for *CDKN2A*, which did not show significant differences.

Our results suggest that the expression of *TP53* downstream target genes in *OSCC* is not influenced by the mutation status of *TP53*, while it correlates with the overall tumor mutation load. *TP53* downstream target genes are also differentially expressed in tumorous versus adjacent margin tissue.

## 4 Discussion

Our study aimed to improve the prognostics of OSCC and to better understand this aggressive and heterogenous group of tumors. Firstly, using a large cohort of patients, we highlighted the performance of high WPOI as a marker of aggressive tumor behavior. We believe this is an important piece of evidence to support inclusion of this parameter in the diagnostic process. Secondly, on a smaller cohort of patients, we wanted to evaluate if the determination of DNA mutations in tumors or surgical margins will serve as a novel biomarker. Our results do support NCCN as a good predictor of relapse and did not sufficiently corroborate DNA mutations in margins as an independent prognostic factor despite the fact that the pathological mutations were detected in several margins.

### 4.1 Significance of morphological features of OSCC

Our study focused on possible associations of DOI in OSCC patients with PNI, WPOI, budding, and metastasis. WPOI turned out to be a key prognostic factor, with higher grades associated with higher DOI and worse overall survival. Similarly, the prognostic role of WPOI and tumor budding has been recently demonstrated by several studies highlighting them as significant risk factors for lymph node metastasis in all stages of OSCC (16, 35). Thus, WPOI can be associated with late metastasis in surgically treated patients (36) and bone invasion, especially for a higher grade of WPOI (WPOI 4–5) (37). In our case, tumor budding negatively correlated with patient survival. Similarly, a greater presence of tumor buds (more than 5 clusters at the invasive front of the tumor) displayed a poor prognosis previously (38). Few studies also examined the association of WPOI and tumor budding with the distance of the tumor from the resection margin. Köhler et al. (39) reported that a safe resection margin in patients with WPOI 1/2/3 was 1.7 mm, while in patients with WPOI 4/5, a higher incidence of local recurrences was observed until a resection margin of 7.8 mm (39). However, Kligerman et al. (40) did not find a statistically significant association of WPOI with recurrence, in contrast to tumor budding (40).

Despite the evidence of the negative impact and prediction of the negative behavior of OSCC described above, the current ICCR guidelines recommend tumor grading and DOI as the main assessment criteria for OSCC, while WPOI and tumor budding are not routinely evaluated. In the current guidelines, the ICCR recommends the evaluation of the invasive front as a cohesive/non-cohesive/dispersed pattern of invasion (21).

One of the potential applications of WPOI is to predict the biological behavior of the tumor and optimize the surgical approach and reduce the probability of local recurrence. For example, a higher grade of biopsy pattern of invasion (BPOI4), which is analogous to WPOI, is directly comparable to the depth of tumor invasion (41). Therefore, we propose that WPOI should be considered an important prognostic factor for OSCC that can complement tumor grading and DOI in the diagnosis and treatment of this disease.

### 4.2 Clinical significance of surgical margins

Clean resection margins during the surgical procedure are an important prognostic factor. Positive resection margin has been repeatedly shown to significantly increase the risk of local tumor recurrence and decrease patient survival, so within the framework of the multidisciplinary team after surgery, second-look resection is recommended whenever it is possible (42–45). However, there is no consensus on what distance from the tumor constitutes a safe resection margin to prevent regional tumor recurrence. Currently, there are two main classifications (ICCR and NCCN) to define resection margins as positive, close, or distant. ICCR and NCCN differ in the definition of “positive margins”, where ICCR considers a margin positive if there is less than 1 mm of tumor-free tissue, while the NCCN considers a margin positive if there is tumor involvement at the inked margin (**Figure 2**). In our cohort, we found that only the positive resection margin according to the NCCN was statistically significantly associated with local tumor recurrence. On the contrary, according to the ICCR, which has a stricter definition of positive margins, we did not find a statistically significant association. Therefore, we propose that instead of using these classifications, it may be more informative to define a safe distance from the tumor to the resection margin, which will significantly correlate with local tumor recurrence. The literature reports various distances as safe margins for OSCC: 1.6 mm (46), 2.2 mm (47), 2.5 mm and 3.5 mm (48), and up to 5 mm (49, 50), which demonstrates how variable the results can be.

### 4.3 Mutation profile in OSCC tumors highlighting potential impact on treatment

Squamous cell carcinoma is marked by diverse, poorly understood molecular mechanisms, despite similar clinical and histological features of tumors due to the genetic heterogeneity of squamous cells (51). Genetic biomarkers can offer a potential for tumor identification, improving diagnosis, staging, and personalized oncological therapy, provided they are specific and sensitive in clinical practice and resistant to the interference from unrelated inflammatory processes (51).

The depth of the tumor invasion at biopsy has been proposed as a predictor of metastatic risk, but this hypothesis was not supported by Dik et al. (9) in their study. Despite this lack of success in their study, the use of markers capable of detecting the risk of tumor recurrence based on the probatory biopsy could play a role in the choice of surgical approach and the extent of resection (9).

A recent publication based on the application of multivariate regression algorithms suitable for higher dimension data identified 3 genes (*MAMDC2*, *SYNPO2* and *ARMH4*) as biomarkers of gene expression signatures for tumor and marginal tissue zones. By this molecular signature, they were able to discriminate tumors and the marginal zones between the close and distant margins, though with very limited power to predict disease recurrence (52). Another work described three other tumor genes (*CCDC66*, *ZRANB2* and *VCPKMT*) for positive margin prediction based on gene expression signature (53).

One of the goals of our study was to define the molecular margins, i.e., to detect possible pro-oncogenic mutations in the tissue that remained after tumor resection, which could help to more accurately predict the possible risk of regional tumor recurrence. In the past, DNA analysis of resection margins with detection of methylation levels in p16, DCC, KIF1A, and EDNRB (54) or analysis of the presence of *TP53* mutation in resection margins (55) have been published. Despite the encouraging results, these analyses have not been introduced into routine clinical practice yet due to their limitations.

Our molecular analysis of tumor-adjacent and tumor tissue revealed several mutations in adjacent tissues; though they cannot serve as independent predictors of disease relapse. Specific DNA mutations in tumors or mutated margins unfortunately did not reveal any associations with the ability to prognose clear margins. In our cohort, we detected mutations in the *TP53* and *CDKN2A* genes in the non-cancer margin tissues that surround the tumor in six patients; these, however, did not seem to be associated with the relapse of the disease in these individuals. Nevertheless, some limitations of our analysis should be considered, in particular the short follow-up period, which may have resulted in insufficient evidence of the association between the presence of mutations and the time to relapse. Other limitations could lie in the sampling site or the small amount of tissue collected, which may not represent the complete tumorous/marginal tissue. In the future, it would be useful to consider the usage of another, more specific method, such as spatial transcriptomics or multi-fluorescent staining, to examine cell population dynamics in resection margins of OSCC.

**Figure 12:**
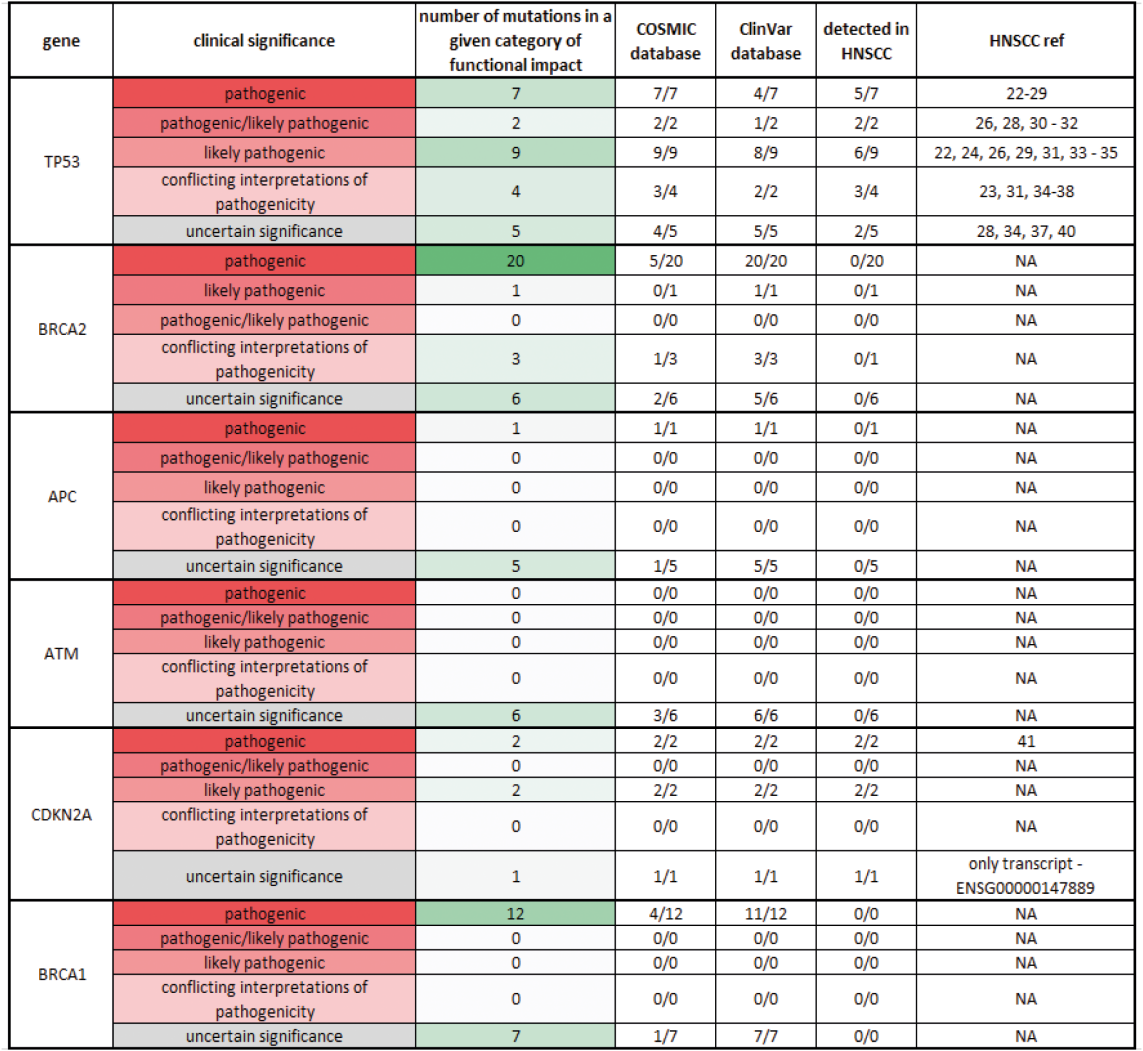
Comparison of the observed mutations in the top six mutated genes in OSCC with the known mutations in the Cosmic and ClinVar databases Observed mutations of the top 6 mutated genes in OSCC: TP53, BRCA2, APC, ATM, CDKN2A, and BRCA1 were searched in Cosmic and ClinVar databases to determine their frequency and clinical significance. The clinical significance was categorized into five levels, from pathogenic to uncertain significance, and color-coded (from red to gray). The number of particular mutations (from none to many) in the group was also color-coded (from white to green). The number of known mutations for each gene in each database was recorded in the corresponding column. The number of observed mutations for each gene in HNSCC was recorded in the following column.

In tumors, we observed high mutation heterogeneity, consistent with previous studies (56–62). To assess the prevalence and significance of the six most frequently mutated genes in our cohort, we searched two databases for their mutation status in other cancer types (**Table S9**). Except for *TP53* and *CDKN2A*, the other four genes (*BRCA1*, *BRCA2*, *APC*, and *ATM*) had not been previously reported to harbor mutations in the head and neck squamous cell carcinoma (HNSCC) (**Figure 12**), although they are well known tumor suppressor genes. *ATM* mutations were mostly related to ataxia-telangiectasia syndrome, a rare genetic disorder (63, 64). *APC* mutations were predominantly studied in colorectal cancer (65–67). The mutations of *BRCA1/2* genes were mostly nonsense substitutions (**Table S9**) that had been mainly associated with breast and ovarian cancer with few exceptions (68–70). Interestingly, our study revealed *BRCA* genes to be among the top mutated genes, which were not detected in the first genomic studies in Asian OSCC populations, but were recently described in Australian and European OSCC populations, though at lower frequencies than in our study (71). This finding warrants further investigation and testing to determine if common *BRCA* (PARP) inhibitors can be of clinical utility in OSCC.

PARP proteins play key roles in various cellular processes, such as chromatin remodeling, replication stress response, and, most importantly, DNA repair. PARP inhibitors block the release of PARP proteins from DNA at the site of the single-strand break, which can interfere with replication fork progression and cause double-strand breaks (DSBs). Cells that lack functional homology recombination repair (HRR) genes (*BRCA1/2* and others) rely on error-prone non-homologous end joining to repair DSBs, which can result in severe genomic instability and cell death. Several PARP inhibitors have been approved by FDA and/or EMA for ovarian cancer in different settings, including olaparib, rucaparib, and niraparib. Recent studies have shown that PARP inhibitors can be beneficial also for patients with somatic mutations in *BRCA1/2* or other HRR genes. Suggestion to extend the utility of PARP inhibitors to other solid cancers such as prostate cancer or small-cell lung cancer has been made (72).

### 4.4 Expression of *TP53* target genes and its association to patient mutation load

*TP53* mutations often result in loss of function, causing the protein to fail to behave as a transcription factor. We have performed an analysis of *TP53* downstream targets to test whether effects of mutated *TP53* differ from those of wild type *TP53* and, thus, if there is some impact on the biology of the tumor. Interestingly, we did not find any significant association between *TP53* mutations and mRNA expression of selected targets: *BAX*, *PUMA, CDKN1A*, and *CDKN2A*. In the light of the recent work by (73) this finding is in line with the HNSCC expression profile. The impact of *TP53* mutations seems to be cancer type and possibly also genomic context dependent (73). It is important to note that the tumors without *TP53* mutations also bore mutations in other cancer-related genes. Further analysis revealed that the total number of tumor mutations influenced the expression of some of these genes, especially *CDKN2A*. *CDKN2A* encodes for p16Ink4a, a tumor suppressor protein involved in cell cycle regulation (74). *CDKN2A* expression is variable and context-dependent in different cancers. As a tumor suppressor gene, its expression is low in many tumors, but, interestingly, its overexpression has also been reported in many cancers, and such high expression of *CDKN2A* may affect the clinical outcome of the patient (75, 76). In HNSCC, p16 expression is lost in 74% of tumors, but its prognostic value is unclear (76, 77). *CDKN2A* was downregulated in the tumors with a high tumor mutation load compared to the tumors with two or fewer mutations.

Then, we focused on the expression of *TP53* targets in the tumor versus tumor margin tissue to search for potential novel biomarkers. The qPCR results indicated that the tumorous OSCC tissue has higher expression of *BAX* and *PUMA*, which are pro-apoptotic genes, and *CDKN1A*, which is a pro-senescence gene, than the non-neoplastic marginal tissue. This contrasts with the common view of cancer as anti-apoptotic and anti-senescence, but it is consistent with previous studies that reported a high expression of *BAX* at the mRNA and protein level and a high degree of apoptosis in oral cancer (78–81). Similarly, we recorded high expression of *PUMA* in the neoplastic tissue, especially in the tumor samples with one or no mutations compared to samples with two or more mutations. This correlates with lower *PUMA* expression in normal non-neoplastic (and non-mutated) tissues. *PUMA* is considered one of the apoptotic activators, and its expression can be regulated by p53-dependent or independent mechanisms (82). In HNSCC, *PUMA* has been reported to decrease with increasing tumor size, and its levels have been found to be lower than in the adjacent non-neoplastic tissue (83), which differs from our findings. As mentioned above, *PUMA* expression is not only p53-dependent but can also be influenced by other factors, such as hypoxia and DNA damage (84). Therefore, overexpression of the pro-apoptotic markers *BAX* and *PUMA* may reflect their alternative functions in neoplastic tissue (85). Nonetheless, the data about *PUMA* expression available in the literature, together with our findings, are still not fully conclusive and warrant further investigation of its role in the carcinogenesis of OSCC.

The expression levels of *CDKN1A* encoding p21, a tumor suppressor protein that plays a key role in DNA damage response and DNA repair (86, 87) was slightly higher in the neoplastic tissue compared to the adjacent marginal tissue, which may correspond to increased DNA damage and an appropriate cellular response in the tumor. This finding is consistent with a previous study showing that patients with HNSCC with high p21 expression have a less favorable prognosis (88).

## 5 Conclusion

In summary, our retrospective study revealed that WPOI is the most important factor predicting tumor recurrence and overall survival. We also defined four groups of patients in our retrospective study, where the group of high-risk patients was defined by WPOI 4 and 5, a tumor located in the floor of the oral cavity, increased budding, higher DOI, and younger age. This group of patients needs special attention as these morphological prognostic factors are associated with negative outcomes. Moreover, we uncovered that only the positive resection margin, according to the NCCN, was statistically significantly associated with local tumor recurrence, while no association was found with classification according to the ICCR. Therefore, we propose that a new definition of a safe distance of the tumor to the resection margin is necessary for future prediction of tumor behavior. Mutations in the genes *TP53* and *CDKN2A* were found in the surgical margins in several patients; however, they did not exhibit significant association with the relapse of the disease. This analysis will need to be performed on a larger cohort of patients as uncovered mutations in tumor suppressor proteins affect cell cycle regulation with a substantial clinical outcome for the patients. High mutation load in tumors is associated with shorter time to relapse and the nature of mutations might also allow better personalization of the subsequent care for patients after surgical removal of OSCC tumors.

## Data availability statement

The datasets presented in this study can be found in online repositories. The names of the repository/repositories and accession number(s) can be found in the article/**Supplementary Material**. Sequencing data is deposited in the EGA database under accession number EGAD50000000106 (https://ega-archive.org/datasets/EGAD50000000106).

## Ethics statement

The studies involving humans were approved by Ethics Committee of the University Hospital Ostrava (No. 514/2018). The studies were conducted in accordance with the local legislation and institutional requirements. The participants provided their written informed consent to participate in this study.

## Author contributions

PH: Conceptualization, Funding acquisition, Writing – original draft, Writing – review & editing, Data curation, Formal analysis, Investigation, Methodology, Visualization. JR: Writing – original draft, Writing – review & editing, Data curation, Formal analysis, Investigation, Methodology, Visualization. ZCh: Writing – original draft, Conceptualization, Methodology. OM: Conceptualization, Writing – original draft, Writing – review & editing, Formal analysis, Investigation, Methodology, Software, Visualization. BM: Formal analysis, Investigation, Visualization, Writing – original draft, Writing – review & editing. ZČe: Writing – review & editing, Formal analysis. TB: Formal analysis, Writing – review & editing. MF: Writing – review & editing, Formal analysis. DG: Validation, Writing – review & editing. MB: Conceptualization, Funding acquisition, Resources, Supervision, Writing – original draft, Writing – review & editing. TŠ: Conceptualization, Methodology, Writing – original draft, Writing – review & editing. JŠ: Conceptualization, Funding acquisition, Supervision, Writing – original draft, Writing – review & editing.
