# Supplementary figures and images for: Enhancing oral squamous cell carcinoma prediction: the prognostic power of the worst pattern of invasion and the limited impact of molecular resection margins
Source: Front Oncol. 2023 Dec 22;13:1287650. doi: 10.3389/fonc.2023.1287650 (PMC10766711; doi:10.3389/fonc.2023.1287650)

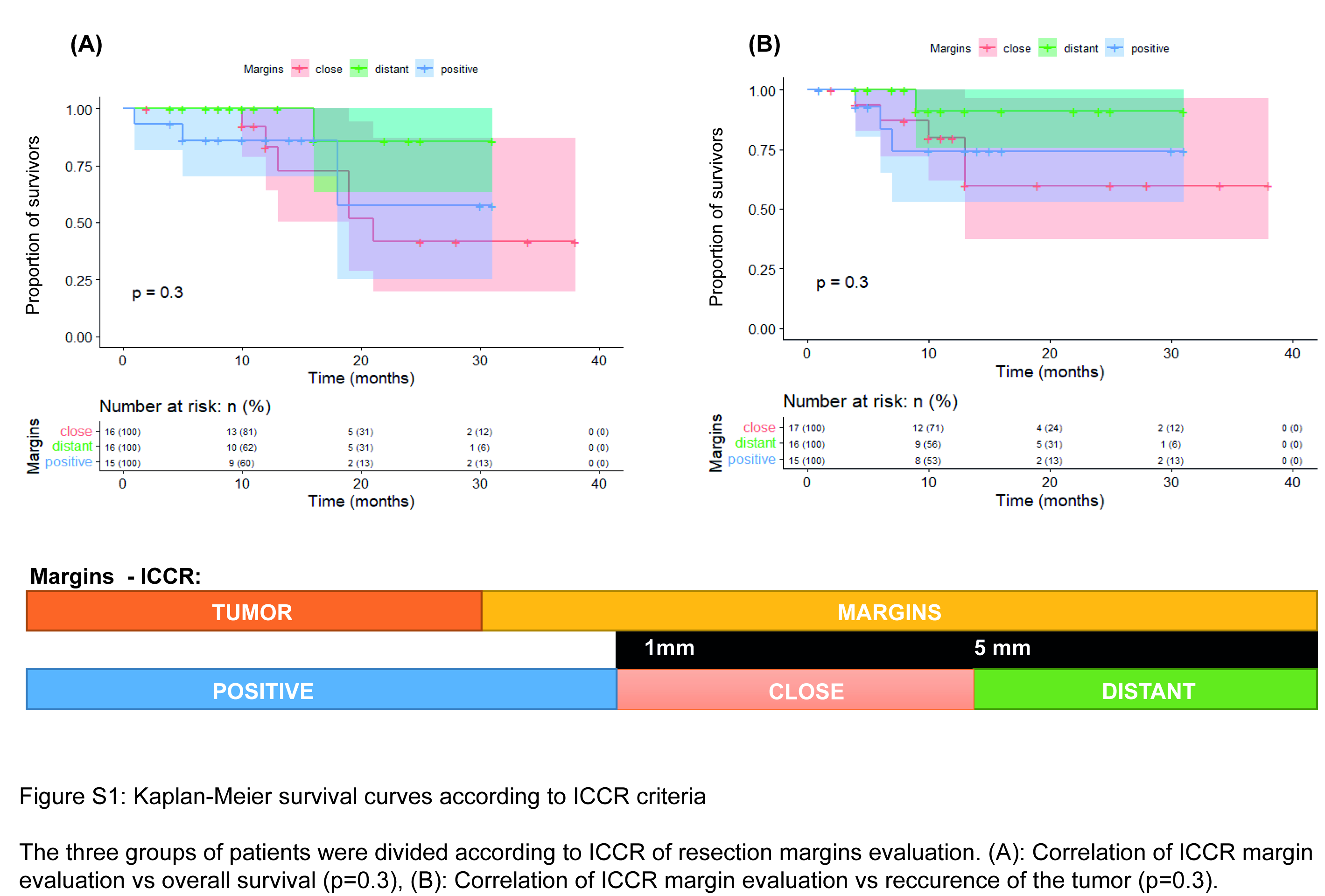

Supplement: Supplementary file 1 [file Image_1.tif]
